# Management of comminuted patellar fractures using suture reduction technique combined with the modified Kirschner-wire tension band

**DOI:** 10.1186/s12893-023-02153-w

**Published:** 2023-08-23

**Authors:** Shenbo Huang, Chang Zou, Guy Romeo Kenmegne, Yijie Yin, Yixiang Lin, Yue Fang

**Affiliations:** 1 Department of Orthopaedics, West China Hospital of Sichuan University, Chengdu, 610041 China; 2 Trauma Center, West China Hospital of Sichuan University, Chengdu, 610041 China

**Keywords:** Comminuted patella fracture, Internal fixation, Modified Kirschner-wire tension band

## Abstract

**Objectives:**

Surgical management of comminuted patella fractures remains a major challenge for the surgeon. We developed a suture reduction (SR) technique to better preserve the comminuted patella. The study aimed to compare the suture reduction technique with conventional reduction (CR) technique in the management of comminuted patellar fractures using the modified Kirschner-wire (K-wire) tension band.

**Methods:**

From May 2016 to September 2020, a total of 75 patients with comminuted patellar fracture were reviewed retrospectively. Among these cases, 35 patients were in the suture reduction group with a mean age of 52 years, while 40 patients were in the conventional reduction group with a mean age of 53 years. All cases were closed fractures. Comminuted patellar fractures were classified as type 34-C3 according to the AO/OTA classification. Radiographs of the knee were obtained at routine follow-up to evaluate the reduction quality and fracture union. Clinical outcomes including range of motion (ROM), visual analog scale (VAS), Lysholm, and Böstman grading scales were measured at the last follow-up. Postoperative complications were also recorded.

**Results:**

The average time from injury to surgery was 5.4 days in suture reduction group and 3.7 days in conventional reduction group (p < 0.05). The surgical time of suture reduction group was less than that of conventional reduction group, but there was no significant difference (p = 0.110) regarding surgical time between the two groups. The average blood loss in suture reduction group was 42.9 ml, while the average blood loss in conventional reduction group was 69.3 ml (p < 0.001). There was no difference regarding fracture union, ROM and knee function score (Lysholm score and Böstman scale) between the two groups. The complication rates were 17.1% in suture reduction group and 12.5% in conventional reduction group respectively (p > 0.05).

**Conclusions:**

In the treatment of comminuted patellar fractures with modified K-wire tension band, the use of suture reduction technique can shorten the surgical time, reduce the surgical trauma, and obtain satisfactory results. This new surgical technique may be particularly effective in management of comminuted patellar fractures when patellectomy would otherwise be considered.

## Introduction

Comminuted patellar fractures comprise 55% of all surgically treated patellar fractures [1]. Comminuted patellar fracture often result from a combination of direct and indirect forces, which may affect the popliteofibular ligaments (PFLs) and retinacula [2]. Because of the crucial function in the extensor mechanism of the knee, patellar fractures require anatomic reduction with stable fixation to allow for earlier rehabilitation [1, 3–5]. The surgical goal of patellar fractures is to reconstruct the extensor mechanism while restoring articular congruency [3]. Although various methods have been described for the treatment of comminuted patellar fractures [6–11], tension band fixation has become the most common method for patellar fractures because of its familiarity and simplicity [12–14].

Given the variety of patellar fracture patterns, there is no currently accepted gold standard surgical technique for the treatment of comminuted fracture patterns. As with all intra-articular fractures, restoration of a stable and congruent articular surface is the general goal in the comminuted patellar fractures [15]. Due to the small and numerous fracture fragments, the conventional reduction for large fracture fragments is somewhat inadequate in the reduction of comminuted patellar fractures.

At the beginning of our approach to this technique, we used a simple and operable reduction technique to treat some comminuted patellar fractures. The complete fracture reduction can be achieved by simply stitching the periosteum of the patella. At present, suture reduction technology has relevant application in some fractures. As early as 1978, Miller et al. [16] proposed that unstable bone fragments can be fixed by suturing torn periosteum when treating comminuted supraorbital fractures. H.S.Song et al. [17] reported the treatment of comminuted fracture of greater tuberosity of humerus with suture bridge reduction and fixation, Michael G. Kogan et al. [18] proposed the technique of suture and fixation of displaced avulsion fracture of tibial intercondylar eminence under arthroscope, and achieved satisfactory results in patients. In most cases, suture reduction is a good strategy for the treatment of comminuted fractures with small fragments. However, there is no more application report in the preservation of comminuted patellar fractures. We learned from this suture reduction technology for avulsion or comminuted fractures; therefore, we graphically defined this technique as SR. Preliminary findings suggested that this reduction technique may help reduce the time of intraoperative reduction of the fracture compared to conventional reduction (CR).

Currently, we have applied this reduction technique to a group of patients with comminuted patellar fractures and followed them for a considerable period of time. Therefore, the purpose of this study was to evaluate and compare the efficacy of SR versus CR in the treatment of comminuted patellar fractures with modified Kirschner-wire (K-wire) tension band.

## Materials and methods

### Subjects

The study was approved by the Institutional Ethical Committee in West China Hospital of Sichuan University and written informed consent was obtained from all patients. We retrospectively identified a series of patients with comminuted patellar fractures who underwent surgery by our team between May 2016 and September 2020. All cases were confirmed by a combination of history, clinical examination, X-ray and CT scan. They were treated with SR or CR combined with the modified K-wire tension band. The inclusion criteria were as follows: (i) modified K-wire tension band fixation; (ii) closed comminuted patella fractures (AO/OTA 34-C3); (iii) time from injury to surgery ≤ 3 weeks; (iv) functional exercise and regular follow-up. Exclusion criteria included: (i) manifestation of pathological fractures and previous history of knee injuries; (ii) open fractures; (iii) multiple fractures; (iv) those patients who were lost in follow-up. If the periosteum around the patella was intact or not severely damaged, SR technique may be considered, otherwise CR technique (such as point-type reduction forceps) may be used.

### Surgical technique

#### Exposure

**Fig. 1:**
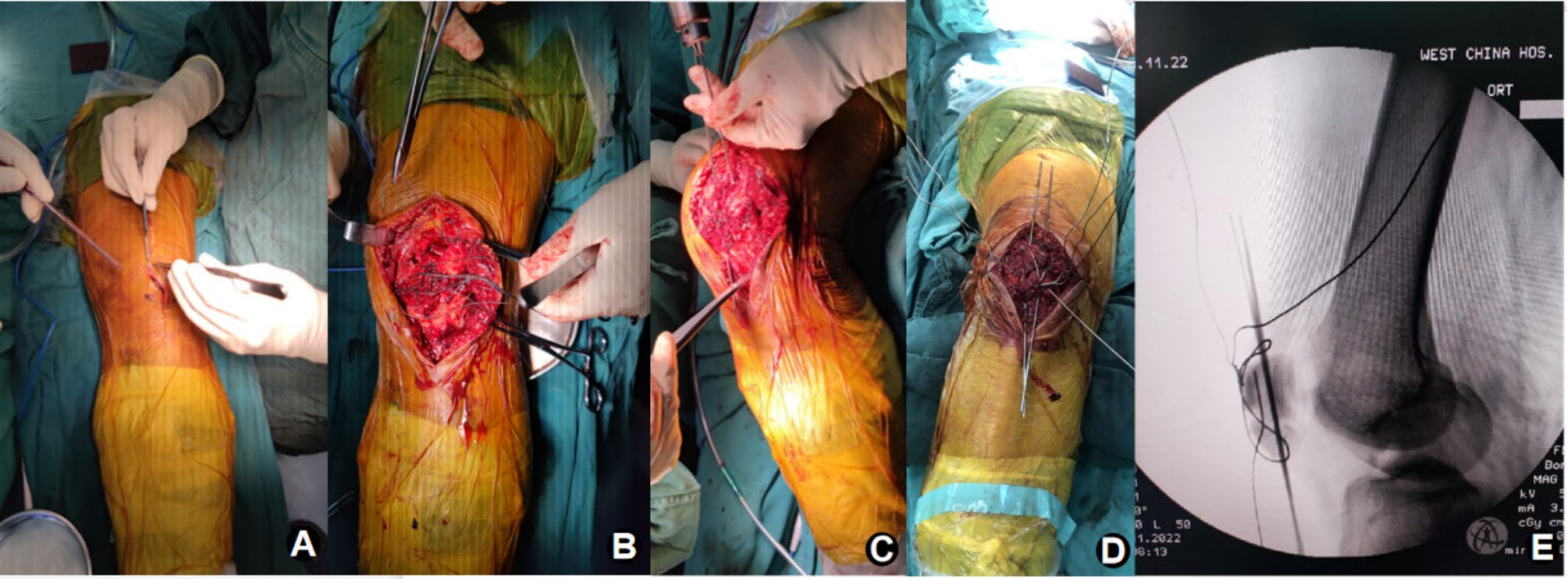
Surgical process

The patient was positioned supine on a radiolucent table with a bump under his ipsilateral hip. A pneumatic tourniquet was routinely applied. A midline longitudinal skin incision of approximately 8–10 cm in length was made above the patella (Fig. 1A). Full-thickness flaps were developed to minimize soft-tissue complications. Blood clots in the fracture margin were debrided, and the torn retinaculum, the fracture pattern, and the fracture surface were identified. After the main fracture site was identified, the fracture hematoma was evacuated.

#### Reduction

##### SR Group

The periosteum was gently pulled and sutured to achieve natural reduction of the fracture fragments. The absorbable suture was then used twice around the patella to increase the strength of the reduction. In this way, the fracture fragments became a whole due to the envelope of the periosteum (Fig. 1B). If the fragments were exposed without periosteum cover, 1.0-mm K-wires or 1.5 mm-screws could be utilized. The reduction was checked radiologically by intraoperative fluoroscopy (Fig. 1C and D) and digital palpation. Care should be taken to protect the periosteum during this process.

##### CR group

A large point-type forceps was used to reduce the main fragment, in which the K-wires could be used as a crowbar tool to assist reduction. Reduction of main fragments was preferred.

#### Fixation and closure

The modified K-wire tension band technique was performed according to the AO Manual of Internal Fixation (Fig. 1E). After fixation, re-fluoroscopy was performed to confirm the quality of reduction. The proximal portion of the wires was trimmed to avoid irritation of the soft tissues. The retinacular tears were repaired with an interrupted absorbable suture. Before closure, the knee was carefully flexed under fluoroscopic guidance to confirm stability of the construct. The remainder of the incision was then sutured in a layered fashion.

### Postoperative rehabilitation

No immobilization was necessary after surgery. Functional exercises were initiated 24 h after the surgery, including active circumduction-movements of ankle and contraction of quadriceps femoris. Passive quadriceps exercises, straight leg raising, and progressive knee flexion were initiated on the fifth postoperative day. Protective weight-bearing was started with crutches on the seventh day after surgery. Full range of motion (ROM) and full weight-bearing without walking assistance started at 4 weeks after surgery. The entire rehabilitation process was checked and modulated through outpatient clinic visits by the operating surgeon.

### Postoperative follow-up and assessment

The patients were followed up regularly at 1, 2, 3, 6 months and one year after surgery and yearly thereafter. Anterior-posterior and lateral radiographs were obtained to assess healing of fractures at routine follow-up intervals. All the patients were examined for ROM with a goniometer. Clinical outcomes included ROM, visual analog scale (VAS), Lysholm knee scale and Böstman scale obtained at the final follow-up by the one of the authors [19, 20].

### Statistical analyses

Statistical analyses were conducted using SPSS 25.0 software (SPSS Chicago, IL, USA). The statistical methods adopted included frequency, percentage (%), mean, t test, Mann-Whitney U test, Fisher’s exact test, and Pearson’s chi-squared test. A value of p < 0.05 was considered as statistically significant difference.

## Results

### Demographics of subjects

All the 75 patients were included in this study. Among the patients, there were 35 patients in the SR group (11 males and 24 females) with a mean age of 52 years (range, 25–74 years), and 40 patients in the CR group (17 males and 23 females) with a mean age of 53 years (range, 20–82 years). All fractures were closed injuries, and there was no statistically significant difference in the general characteristics of the two groups (Table 1).

**Table 1:** The demographics of subjects

Variable	SR	CR	p value
**Mean age (years)**	52 (25–74)	53 (20–82)	0.979
**Gender**			0.323
Male	11 (31.4%)	17 (42.5%)	
Female	24 (68.6%)	23 (57.5%)	
**Body mass index (BMI)**	22.3	23.4	0.086
**Visual Analogue Scale (VAS)**	3.5	3.9	0.064
**Smoking**			0.930
Yes	5 (14.3%)	6 (15.0%)	
No	30 (85.7%)	34 (85.0%)	
**Marital status**			0.312
Married	31 (88.6%)	32 (80.0%)	
Unmarried or divorced	4 (11.4%)	8 (20.0%)	
**Side of injury**			0.877
Right	19 (54.3%)	21 (52.5%)	
Left	16 (45.7%)	19 (47.5%)	
**Mechanism of injury**			0.588
Motor vehicle collision	1 (2.9%)	3 (7.5%)	
Fall from height	1 (2.9%)	2 (5.0%)	
Slip	33 (94.2%)	35 (87.5%)	
**Total**	35 (100%)	40 (100%)	-

### Surgical-related parameters

The average blood loss in SR group was 42.9 ml, while the average blood loss in CR group was 69.3 ml (p < 0.001). The mean surgical time was longer in CR group as compared to SR group, but there was no significant difference (p = 0.110) between the two groups. None of the patients received blood transfusion. There was no significant difference (p = 0.08) in the fracture union time (10.1 weeks for SR group vs. 10.7 weeks for CR group). The mean ROM of the two groups were 122 degrees and 118 degrees, respectively. During postoperative follow-up, the Lysholm score was 91.8 and 91.3 respectively in the SR and CR group, with no statistically significant difference (p = 0.991); The Böstman scale in the SR group was 27.4 and 27.2 in the CR group, with no statistically significant difference (p = 0.211).

### Complications

The complication rates were 17.1% in SR group (6 cases) and 12.5% in CR group (5 cases) (p = 0.746) (Table 2). With the exception of one patient whose internal fixation was removed due to migration of the K-wires, all patients with complications refused reoperation.

**Table 2:** Perioperative and follow-up parameters

Variable	SR	CR	p value
**Mean time to surgery (days)**	5.4 (0–16)	3.7 (0–12)	0.016
**Surgical time (minutes)**	63.6 (45–105)	71.9 (45–120)	0.110
**Blood loss (mL)**	42.9	69.3	< 0.001
**Blood transfusion**			-
Yes	0 (0%)	0 (0%)	
No	35 (100%)	40 (100%)	
**Fracture union (weeks)**	10.1	10.7	0.08
**Postoperative complications**			0.746
Limited flexion	1 (2.9%)	1 (2.5%)	
Extension lag	0 (0%)	1 (2.5%)	
Knee stiffness	1 (2.9%)	1 (2.5%)	
Anterior knee pain	2 (5.7%)	1 (2.5%)	
K-wires migration	2 (5.7%)	1 (2.5%)	
**Range of motion (ROM)**			-
Degree of extension (mean)	0	0	
Degrees of flexion (mean)	122	118	
**Knee function score**			
Lysholm score (mean)	91.8	91.3	0.991
Böstman scale (mean)	27.4	27.2	0.211
**Follow-up time (months)**	35.7	31.8	0.212

## Discussion

**Fig. 2:**
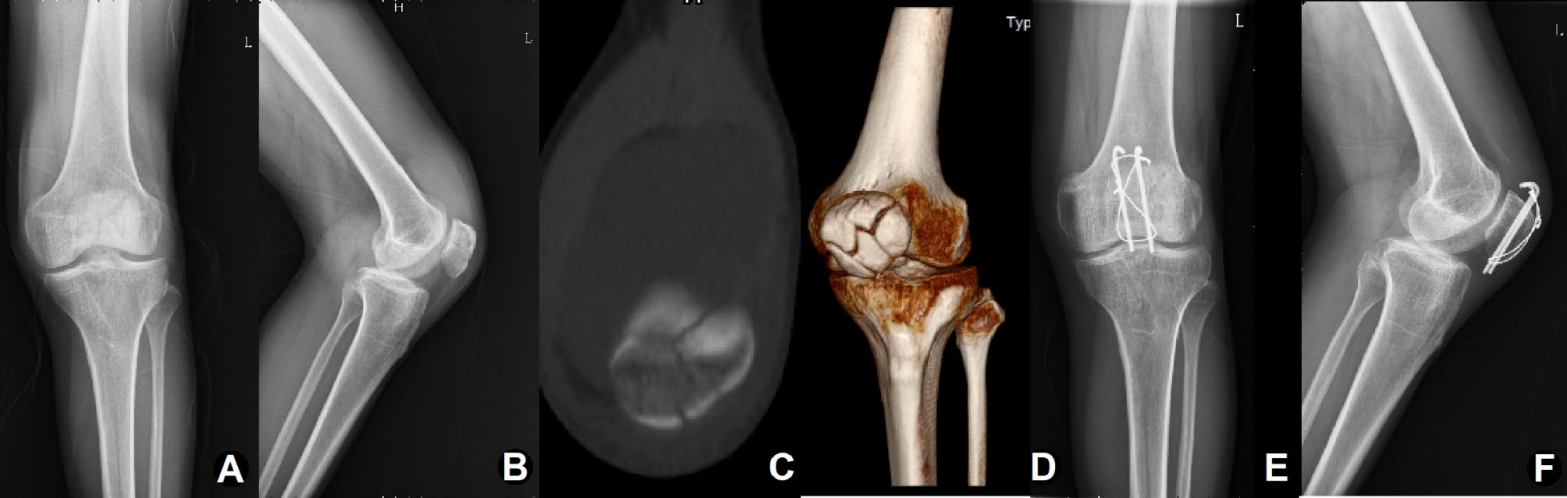
Case example of comminuted patellar fracture (Male, 43 years old, left side) using the suture reduction combined with the modified K-wire tension band. (**A, B**) Preoperative X-ray indicated displaced comminuted patellar fractures. (**C, D**) The regular follow-up at 1.5 years after operation showed fracture union without displacement of internal fixation and (**E, F**) the internal fixation was removed two years after surgery

**Fig. 3:**
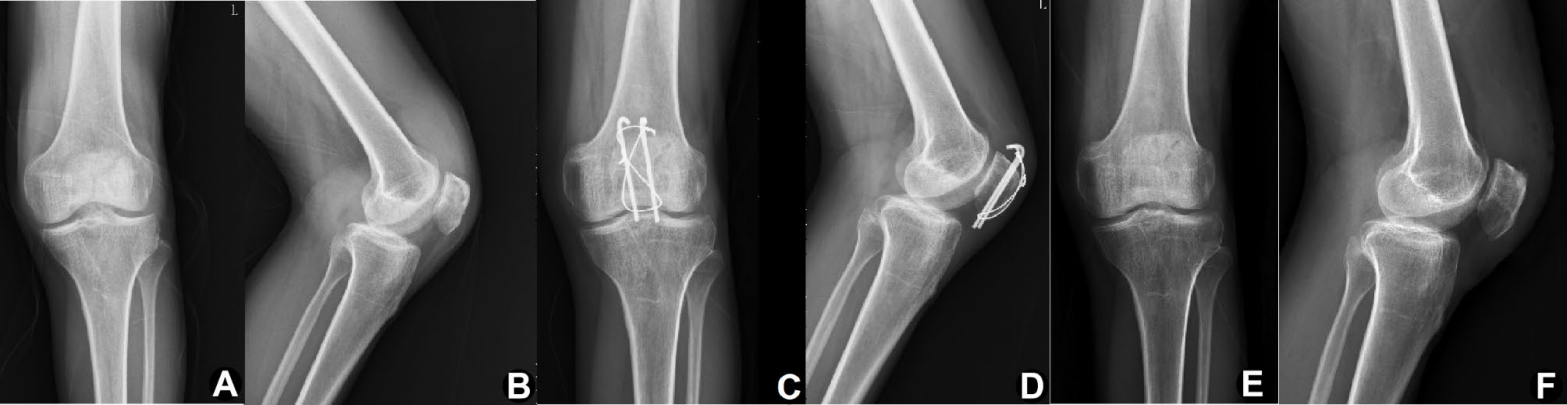
Case example of comminuted patellar fracture (male, 51 years old, left side) using the suture reduction combined with the modified K-wire tension band. (**A, B**) Preoperative X-ray and (**C, D**) CT images indicated displaced comminuted patellar fractures. (**E, F**) The regular follow-up at 1.5 years after operation showed the fracture healed without fixation failure

The purpose of this study is to evaluate and compare the efficacy of SR and CR in the treatment of comminuted patellar fracture with modified Kirschner-wire tension band (Fig. 2**and** Fig. 3). Overall, SR and CR showed good clinical efficacy, low incidence of complications, and high patient satisfaction in the treatment of comminuted patellar fractures with modified Kirschner-wire tension band. Herein, the surgical time and blood loss in the SR group were less than those in the CR group, which could be explained by the following points: Firstly, during the SR process, we mainly sutured the periosteum to restore the integrity of the patella, without excessive dissection of the fracture, thus reducing tissue bleeding. Secondly, preoperative attention to the characteristics of fracture and intraoperative protection of periosteum integrity were conducive to the implementation of SR technique and shorten the operation time. Finally, SR did not rely too much on some reduction tools, such as reduction forceps, K-wires, etc. and the process was relatively simple.

In addition, the difference in surgical time between the two groups was not statistically significant, which may be related to the small sample size. In our series, fracture union was achieved in SR group at an average of 10.1 weeks. Full range of motion (knee flexion/extension) was achieved in SR group except 2 cases with limited knee flexion.

We compared the results of this study with the results obtained by other scholars [8, 10, 11, 21–23]. In those studies, the majority of patients achieved union with full ROM. Compared with the results of those studies and CR group, there were decrease in surgical time as well as the amount of blood loss in our SR technique, which was consistent with the current concept of minimally invasive surgery. Functional results of SR group and other case series using different fixation techniques were summarized in Table 3.

**Table 3:** Different studies reporting on functional outcomes of comminuted patella fractures

	Singer et al.^5^	Suh et al.^7^	Cho et al.^8^	Gao et al.^21^	Sun et al.^22^	Wang et al.^23^	SR Group
**Case (n)**	9	13	30	16	38	25	35
**Fracture type ( AO/OTA** **classification)**	34-C3	12 cases: 34-C3; One case: 34-C1	25 cases 34-C3; 5 cases 34-C2	No mention	28 cases: 34-C3; 10 cases: 34-C2	6 cases: 34-C3; 19 cases: 34-C2	34-C3
**Closed fractures (n)**	9	11	27	16	38	25	35
**Fixation method**	Low profile mesh plate	Headless compression screws with additional separate vertical wiring	Miniplate augmented tension-band wiring (TBW)	A miniature plate with a tension band wire	Modified cerclage wiring	Modified anterior ellipsoidal cap titanium cable tension band	suture reduction combined with the modified Kirschner-wire tension band
**follow-up (months)**	19.6 (12–33)	16 (12–25)	20 (12–28)	15.6 (12–20)	16.1 (6–36)	25 (17–39)	35.7 (6–64)
**Surgical time (minutes)**	69 (55–90 )	No mention	71.1 (60–79)	No mention	66.4 (55–80)	No mention	63.6 (45–105)
**Postoperative complications**	One case superficial wound issues; One case deep venous thrombosis (DVT)	Four cases thigh muscle wasting; Three cases mild anterior knee pain; Six cases removal of hardware	One case postoperative infection; Four cases implant removal; Four cases flexion contracture; Three cases extension lag	No	No	Two cases minor complications (soft tissue irritation, cellulitis)	One case limited flexion; One case knee stiffness; Two cases anterior knee pain; Two cases K-wires migration
**Range of motion (ROM)**	Eight cases: full range of knee flexion/extension; One case:Extension:-10 degrees, flexion: 90 degrees	134.2 degrees (120–145)	120 degrees (110–130)	All cases: full range of knee flexion/extension	130 degrees (110–140)	No mention	122 degrees (60–135)
**Knee function score**	Lysholm score: 89.1(82–95); Böstman scale: 27.2(22–30).	Lysholm score: 94.4 (84–100); Böstman scale: 28.7 (25–30)	Lysholm score: 94.3 (82–100); Böstman scale:28.6(26–30)	Lysholm score: 91.6(84–97); Böstman scale: 26.4(22–30)	Böstman scale: 28.7 (20–30)	Böstman scale: 27.3 (23–30)	Lysholm score: 91.8(65–100); Böstman scale: 27.6(13–30).

Symptomatic implant is one of the reasons why many surgeons have abandoned the K-wire tension band construct for patella procedures [12, 24–26]. Hoshino et al. [12] reported symptomatic hardware removal in 36.8% of patients treated by K-wires and cerclage. In another retrospective study, rate of implant prominence and subsequent removal was 13%. Most irritation was usually associated with the proximal end of the K-wire when the wire initially fails to be imbedded deeply under the soft tissue or was displaced due to loss of anchoring [27]. In the current study, owing to our efforts to reduce implant prominence on the anterior cortex, three cases of hardware complications were reported; one of our approaches was the development of one-layered soft-tissue flaps along the surgical incision; in our opinion, this may lead to low rate of implant prominence.

The goal in treating comminuted patellar fractures is to restore the extensor mechanism, anatomically reduce the articular surface, and provide a stable construct to allow early rehabilitation [3, 28, 29]. In order to achieve this goal, fixation constructs should be versatile, stable and strong enough to allow early mobilization. Recent biomechanical and technical studies have shown that plate internal fixation for comminuted patellar fractures is successful [9]. While various techniques of internal fixation have been recommended [6–11], a gold standard of treatment has not been established. In addition, many severely comminuted patellar fractures are commonly treated with partial or total patellectomy, which results in devastating outcomes [30]. These facts indicate that management of comminuted patella fractures remains challenging.

Surgical fixation of articular fragments in the comminuted patellar fractures is often too complex and difficult because of inherent weakness of the bone and relatively small fracture fragments. In the treatment of such fractures, the modified tension band wiring fixation alone is often unable to achieve effective fixation. Yang et al. [6] suggested the management of displaced comminuted patellar fracture with titanium cable cerclage. In this approach, the articular fragments were not fixed directly, and the reduction was maintained only by the cerclage; failure may develop during the rehabilitation due to a lack of direct fixation to the articular fragments. The modified tension band technique can be used in conjunction with the cerclage to enhance the stability of the fixation. However, circumferential cerclage may also result in multiple segmentation of the wire. We applied a reduction technique for comminuted patellar fractures, which was based upon suture reduction concept, this suture reduction technique has been applied to some comminuted fractures with small fragments, but there is no more application report in the preservation of comminuted patellar fractures.

The limitations of our study were the small sample size, retrospective nature, and the lack of long-term follow-up. For the treatment of comminuted patellar fractures, biomechanical analysis would also be beneficial. Additional prospective and biomechanics studies should be conducted to confirm these outcomes in the future.

## Conclusion

Suture reduction combined with the modified K-wire tension band is a good option in management of comminuted patellar fractures with good clinical outcomes. Although our study was limited by its small sample size, we believed that our technique may be particularly useful for the comminuted patellar fractures which would traditionally be treated with patellectomy.

## Data Availability

The datasets generated during the current study are available from the corresponding author on reasonable request.
